# Supplementary material for: Improving the informativeness of Mendelian disease-derived pathogenicity scores for common disease
Source: Nat Commun. 2020 Dec 7;11:6258. doi: 10.1038/s41467-020-20087-2 (PMC7721881; doi:10.1038/s41467-020-20087-2)
Supplement: Supplementary file 1 — Supplementary Information [file 41467_2020_20087_MOESM1_ESM.pdf]

Supplementary Information for Kim et al.: Improving the informativeness of Mendelian disease-derived pathogenicity scores for common disease

Supplementary Tables

Supplementary Table 1. List of 41 independent diseases and complex traits analyzed. Analogous to a previous study[1], we considered 89 GWAS summary association statistics, including 34 traits from publicly available sources and 55 traits from the UK Biobank (up to N = 459K); summary association statistics were computed using BOLT-LMM v2.3[2,3]. We obtained 41 independent traits (average N = 320K) with genetic correlation less than 0.9 (computed using cross-trait LDSC[4]). For 6 traits, we analyzed two different sources (both publicly available and UK Biobank), resulting in total 47 summary statistics analyzed. For each trait, we report a trait identifier, trait description, reference, sample size, and heritability z-score.

Trait identifier	Trait description	Reference	N	h_g^2	z
PASS_AgeFirstBirth	Age first birth	Barban et al., 2016 Nat. Genet.[5]	222,037	0.062	15.220
PASS_NumberChildrenEverBorn	Number children ever born	Barban et al., 2016 Nat. Genet.[5]	318,863	0.022	9.250
PASS_Anorexia	Anorexia	Boraska et al., 2014 Mol. Psych.[6]	32,143	0.241	8.489
PASS_Crohns_Disease	Crohn's Disease	Jostins et al., 2012 Nature[7]	20,883	0.495	8.327
PASS_Ulcerative_Colitis	Ulcerative Colitis	Jostins et al., 2012 Nature[7]	27,432	0.260	6.411
PASS_Height1	Height	Lango Allen et al., 2010 Nature[8]	131,547	0.221	17.680
PASS_Type_2_Diabetes	Type 2 Diabetes	Morris et al., 2012 Nat. Genet.[9]	60,786	0.090	6.599
PASS_Rheumatoid_Arthritis	Rheumatoid Arthritis	Okada et al., 2014 Nature[10]	37,681	0.181	7.220
PASS_DS	Depressive symptoms	Okbay et al., 2016 Nat. Genet.[11]	161,460	0.043	8.212
PASS_Years_of_Education2	Years of Education	Okbay et al., 2016 Nature[11]	328,917	0.112	27.975
PASS_Autism	Autism Spectrum	PGC Cross-Disorder Group, 2013 Lancet[12]	10,263	0.610	6.518
PASS_Coronary_Artery_Disease	Coronary Artery Disease	Schunkert et al., 2011 Nat. Genet.[13]	77,210	0.074	6.125
PASS_Schizophrenia	Schizophrenia	SCZ Working Group of the PGC, 2014 Nature[14]	70,100	0.464	18.726
PASS_BMI1	BMI	Speliotes et al., 2010 Nat. Genet.[15]	122,033	0.145	15.538
PASS_Ever_Smoked	Ever Smoked	TAG Consortium, 2010 Nat. Genet.[16]	74,035	0.085	7.870
PASS_HDL	HDL	Teslovich et al., 2010 Nature[17]	97,749	0.119	10.000
PASS_LDL	LDL	Teslovich et al., 2010 Nature[17]	93,354	0.101	8.487
UKB_460K.body_HEIGHTz	Height	UK Biobank[3]	458,303	0.673	26.000
UKB_460K.bmd_HEEL_TSCOREz	Heel T Score	UK Biobank[3]	445,921	0.351	20.283
UKB_460K.blood_PLATELET_COUNT	Platelet Count	UK Biobank[3]	444,382	0.343	21.298
UKB_460K.body_BMIz	BMI	UK Biobank[3]	457,824	0.280	39.943
UKB_460K.lung_FEV1FVCzSMOKE	Forced Vital Capacity (FVC)	UK Biobank[3]	371,949	0.274	24.230
UKB_460K.blood_RED_COUNT	Red Blood Cell Count	UK Biobank[3]	445,174	0.260	21.992
UKB_460K.repro_MENARCHE_AGE	Age at Menarche	UK Biobank[3]	242,278	0.255	23.182
UKB_460K.lung_FVCzSMOKE	FEV1-FVC Ratio	UK Biobank[3]	371,949	0.232	32.662
UKB_460K.bp_SYSTOLICadjMEDz	Systolic Blood Pressure	UK Biobank[3]	422,771	0.229	27.963
UKB_460K.blood_EOSINOPHIL_COUNT	Eosinophil Count	UK Biobank[3]	439,938	0.223	19.884
UKB_460K.body_BALDING1	Balding Type I	UK Biobank[3]	208,336	0.223	14.651
UKB_460K.blood_WHITE_COUNT	White Blood Cell Count	UK Biobank[3]	444,502	0.221	28.282
UKB_460K.blood_RBC_DISTRIB_WIDTH	Red Blood Cell Distribution Width	UK Biobank[3]	442,700	0.215	18.034
UKB_460K.body_WHRadjBMIz	Waist-hip Ratio	UK Biobank[3]	458,417	0.170	24.257
UKB_460K.pigment_HAIR	Hair Color	UK Biobank[3]	452,720	0.166	7.598
UKB_460K.cov_EDU_YEARS	College Education	UK Biobank[3]	454,813	0.139	34.625
UKB_460K.mental_NEUROTICISM	Neuroticism	UK Biobank[3]	372,066	0.115	29.462
UKB_460K.repro_MENOPAUSE_AGE	Age at Menopause	UK Biobank[3]	143,025	0.113	11.968
UKB_460K.other_MORNINGPERSON	Morning Person	UK Biobank[3]	410,520	0.106	25.878
UKB_460K.cov_SMOKING_STATUS	Smoking Status	UK Biobank[3]	457,683	0.102	29.882
UKB_460K.disease_ALLERGY_ECZEMA_DIAGNOSED	Eczema	UK Biobank[3]	458,699	0.083	14.596
UKB_460K.pigment_TANNING	Tanning	UK Biobank[3]	449,984	0.082	6.430
UKB_460K.pigment_SKIN	Skin Color	UK Biobank[3]	453,609	0.082	6.074
UKB_460K.pigment_SUNBURN	Sunburn Occasion	UK Biobank[3]	344,229	0.075	9.313
UKB_460K.disease_RESPIRATORY_ENT	Respiratory and Ear-nose-throat Diseases	UK Biobank[3]	459,324	0.056	14.737
UKB_460K.disease_HYPOTHYROIDISM_SELF_REP	Hypothyroidism	UK Biobank[3]	459,324	0.054	14.622
UKB_460K.disease_HI_CHOL_SELF_REP	High Cholesterol	UK Biobank[3]	459,324	0.051	12.949
UKB_460K.disease_T2D	Type 2 Diabetes	UK Biobank[3]	459,324	0.047	15.533
UKB_460K.disease_DERMATOLOGY	Dermatologic Diseases	UK Biobank[3]	459,324	0.012	6.316
UKB_460K.disease_AID_SURE	Auto Immune Traits (Sure)	UK Biobank[3]	459,324	0.010	6.125

Supplementary Table 2. Expected heritability enrichments of binary annotations derived from boosted Mendelian disease-derived missense scores with significantly negative τ^*. We report the heritability enrichment that is expected based on the boosted annotation's overlap with the baseline-LD model and corresponding published annotations, by assuming that the τ of the annotation is zero. In general, expected enrichments were significantly less than observed enrichments.

Annotations	Prop. SNPs	Enrich.	Enrich. s.e.	Enrich. P	Expected enrich.	Expected enrich. s.e.	Expected enrich. P
REVEL_boosted	0.26%	4.652	0.745	2.72E-05	8.016	0.813	5.01E-23
PolyPhen-2_boosted	0.17%	6.164	0.648	1.47E-07	9.287	0.722	3.10E-32
SIFT 4G_boosted	0.22%	0.570	0.534	3.09E-01	1.733	0.748	3.30E-02
PolyPhen-2-HVAR_boosted	0.18%	6.946	0.535	5.93E-11	8.864	0.800	2.10E-46
PROVEAN_boosted	0.54%	2.402	0.374	7.42E-03	8.487	0.598	7.58E-64
MetaSVM_boosted	1.13%	4.946	0.341	4.70E-19	2.874	0.234	9.18E-53
MetaLR_boosted	0.09%	2.235	0.231	1.04E-04	2.348	0.168	4.44E-67

Supplementary Figures

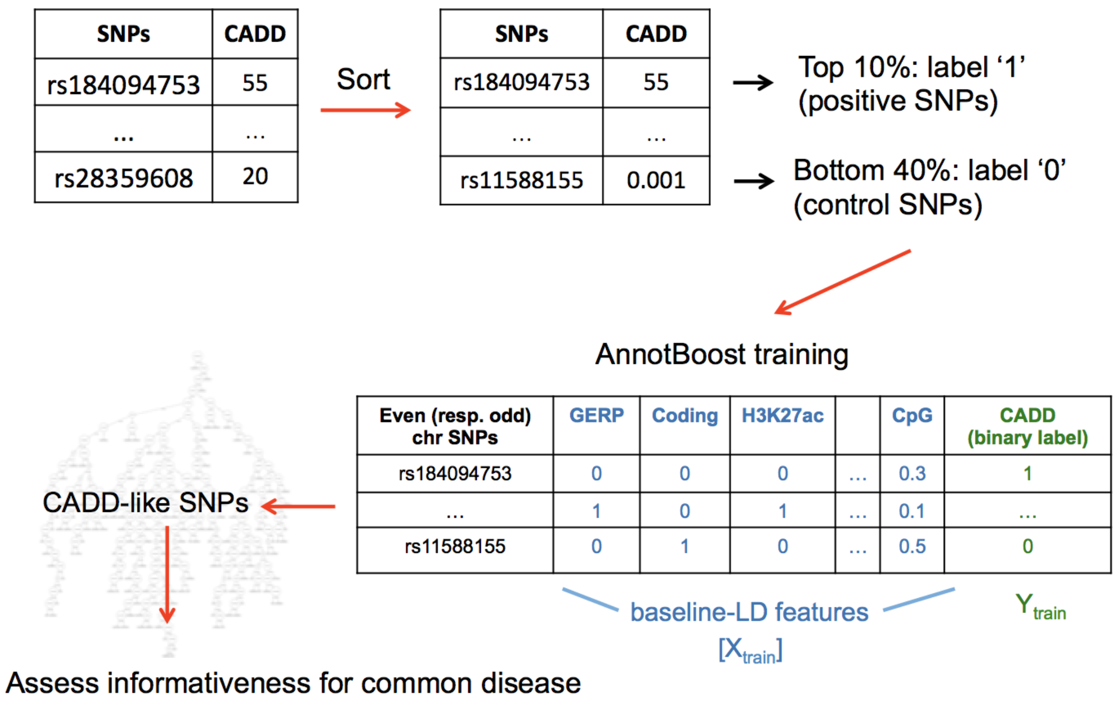

Supplementary Figure 1. Overview of AnnotBoost framework. We describe the AnnotBoost model training. AnnotBoost requires only one input, a pathogenicity score to boost, and generates a genome-wide (probabilistic) boosted pathogenicity score. From the input pathogenicity score (e.g. CADD as shown here), we built a classification model, each for even and odd chromosome SNPs using 75 baseline-LD annotations[18,19] as features. We assessed informativeness of annotations derived from published scores (input) and boosted scores (output) for common disease using S-LDSC[20].

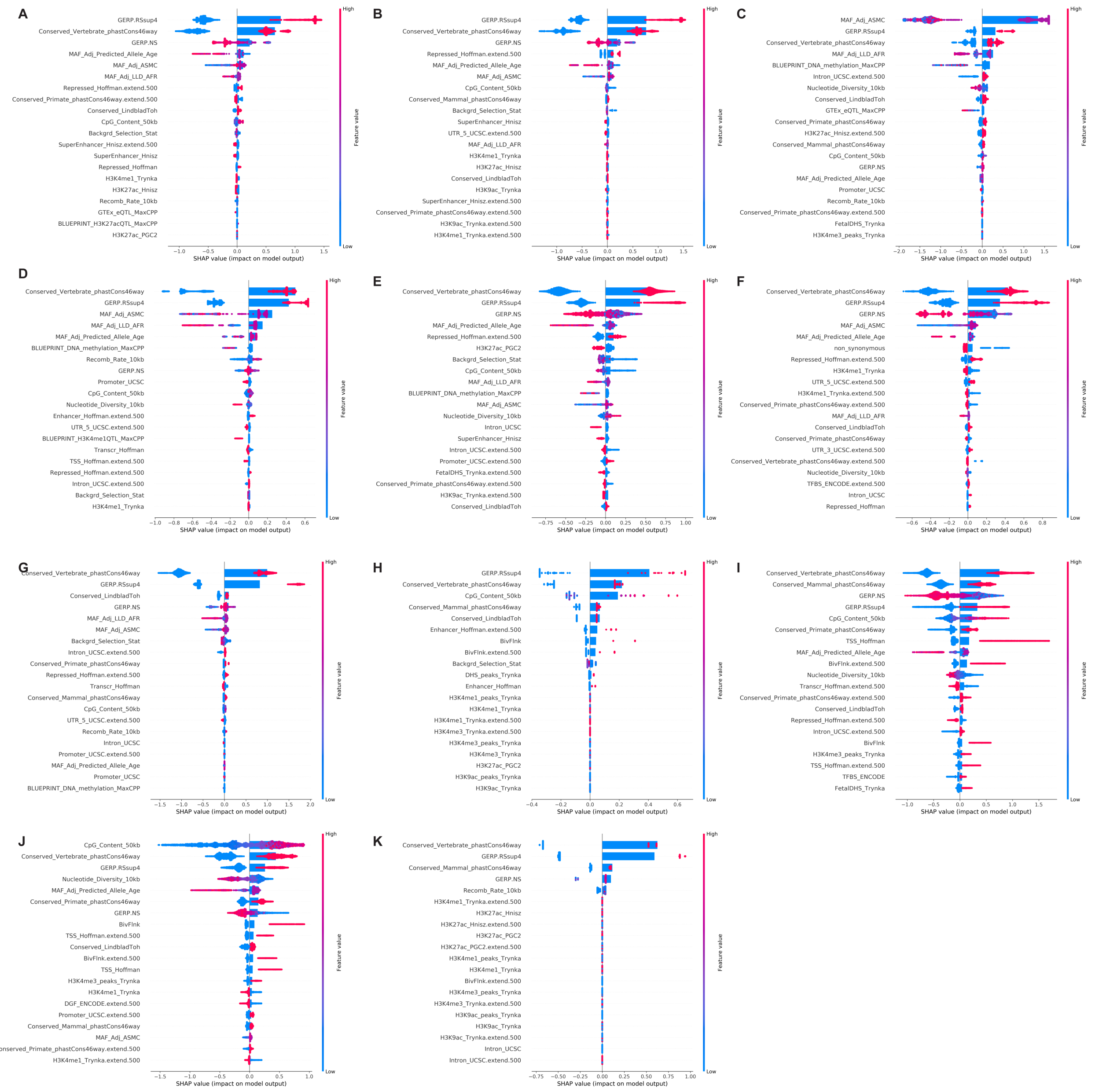

Supplementary Figure 2. Feature importance of boosted Mendelian disease-derived missense pathogenicity scores. We applied SHAP[21] to assess which features from the baseline-LD drives the prediction of 11 boosted missense scores by AnnotBoost. We report the signed impact of top 20 features for each of 11 predictive models: (A) PolyPhen-2, (B) PolyPhen-2-HVAR, (C) MetaLR, (D) MetaSVM, (E) PROVEAN, (F) SIFT 4G, (G) REVEL, (H) M-CAP, (I) Primate-AI, (J) MPC, and (K) MVP. We obtained similar results for even/odd chromosome classifiers; we report odd chromosome results here (see full results online; see URLs).

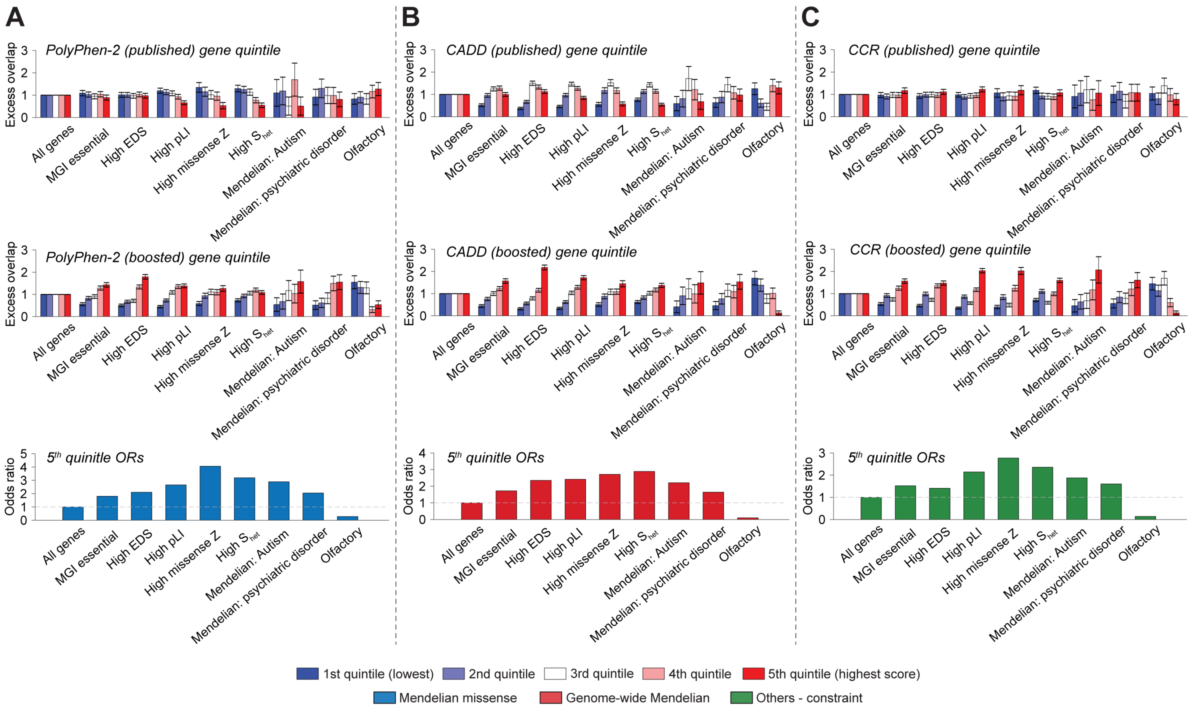

Supplementary Figure 3. Excess overlap between gene scores derived from input pathogenicity scores and 165 reference gene sets of biological importance. We report the excess overlap of genes linked to published and boosted scores in existing gene sets of biological importance (summarized in Supplementary Data 8): (A) PolyPhen-2 [22,23] gene quintiles from published and boosted scores, (B) CADD [24,25] gene quintiles from published and boosted scores, and (C) CCR [26] gene quintiles from published and boosted scores. Error bars represent 95% confidence intervals. Numeric results for excess overlap and correlaton among gene scores are shown in Supplementary Data 9. Numeric results for odds ratios and p-values from Fisher's exact test (two-sided) between published gene quintiles and boosted gene quintiles are reported in Supplementary Data 10.

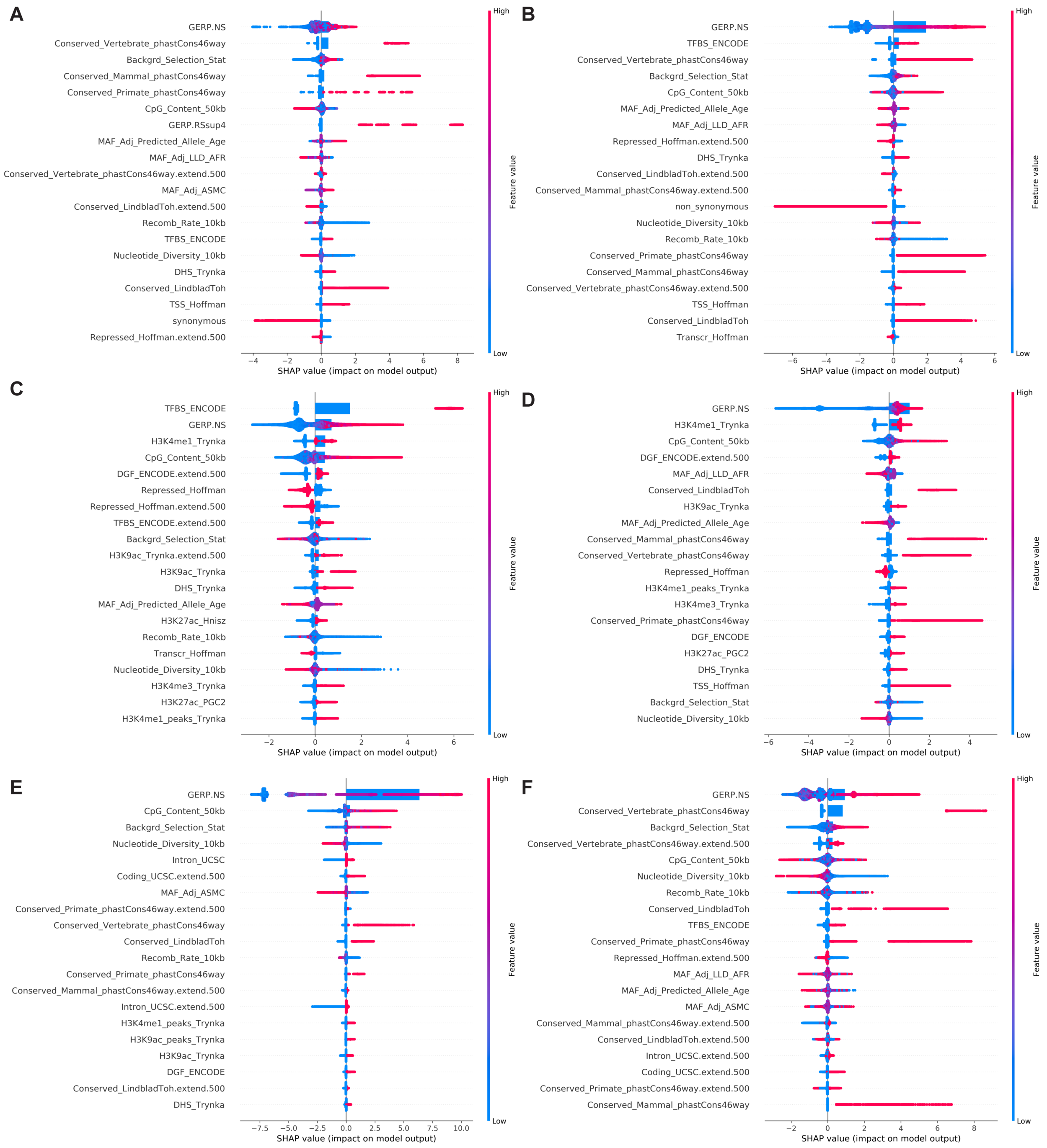

Supplementary Figure 4. Feature importance of boosted genome-wide Mendelian disease-derived pathogenicity scores. We applied SHAP [21] to assess which features from the baseline-LD drives the prediction of 11 boosted missense scores by AnnotBoost. We report the signed impact of top 20 features for each of 6 genome-wide Mendelian disease-derived pathogenicity scores: (A) CADD, (B) Eigen, (C) Eigen-PC, (D) ReMM, (E) NCBoost, and (F) ncER. We obtained similar results for even/odd chromosome classifiers; we report odd chromosome results here (see full results online; see URLs).

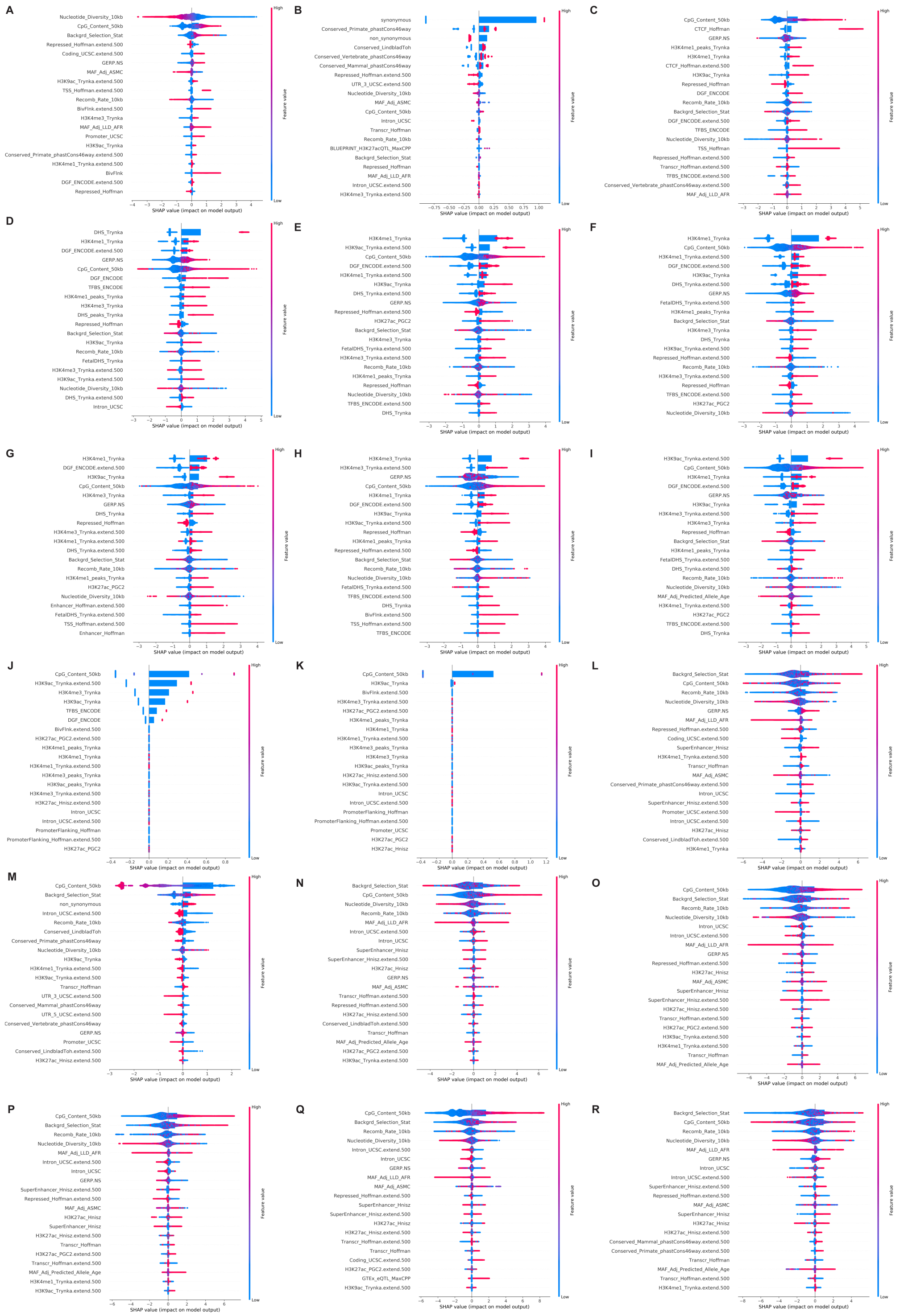

Supplementary Figure 5. Feature importance of boosted scores derived from 18 additional genome-wide scores and 47 baseline-LD model annotations. We applied SHAP[21] to assess which features from the baseline-LD drives the prediction of 18 boosted additional scores by AnnotBoost. We report the signed impact of top 20 features for each of 18 additional scores: (A) CDTS, (B) CCR, (C-I) DeepSEA-CTCF, -DNase, -H3K27ac, -H3K4me1, -H3K4me2, -H3K4me3, -H3K9ac, (J-K) DIS-DNA, -RNA, (L) pLI, (M) LIMBR, (N-Q) Gene network connectivity-Saha, Greene, InWeb, Sonawane, (R) EDS. We obtained similar results for even/odd chromosome classifiers; we report odd chromosome results here (see full results online; see SHAP results of 47 boosted baseline-LD scores online; see URLs).

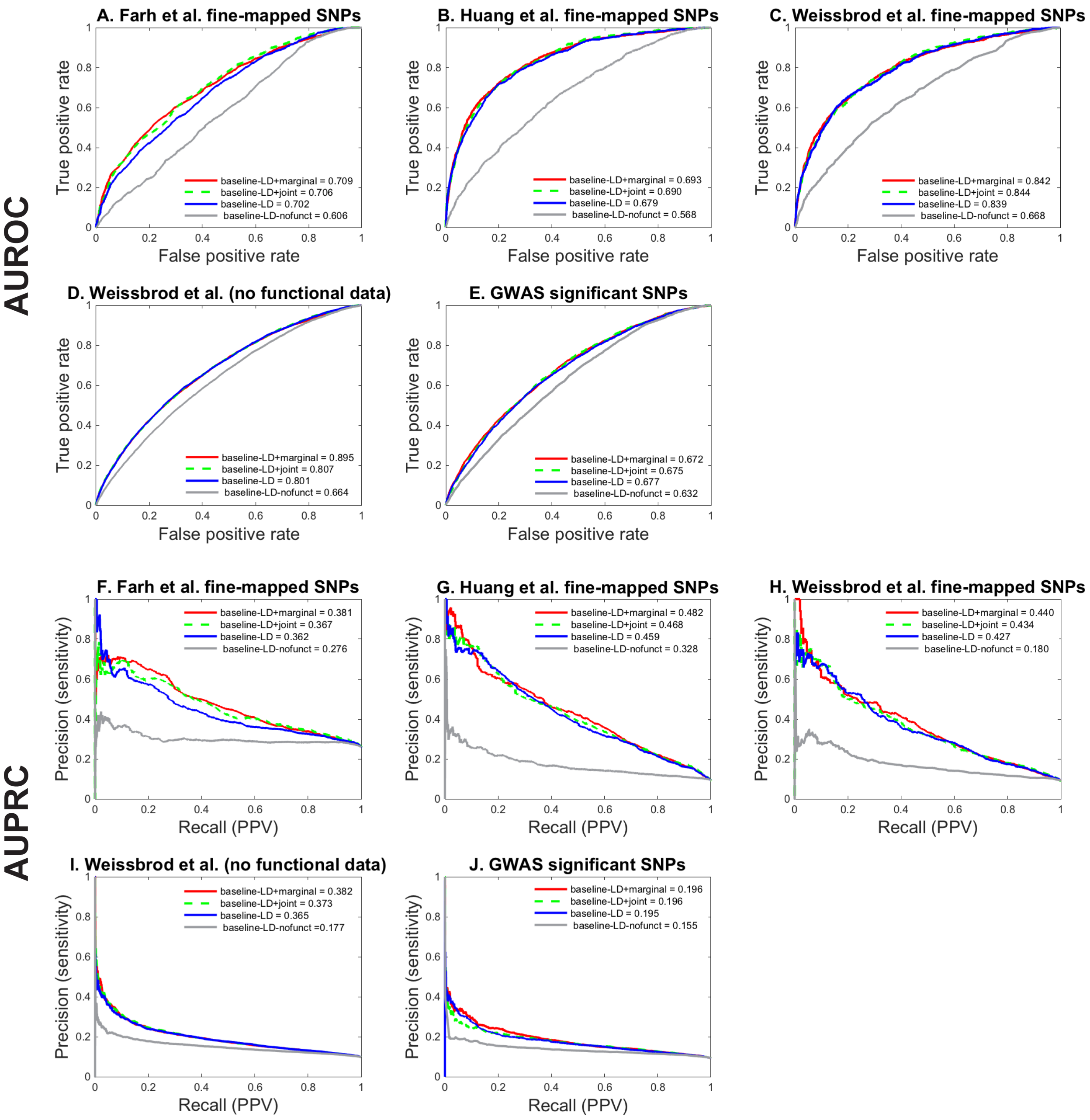

Supplementary Figure 6. Classification of fine-mapped disease SNPs using aggregated scores. We report the true positive rate, false positive rate, precision, and recall along with the classification accuracy (AUPRCs and AUROCs) of four aggregated scores on classifying 5 different independent SNP sets: (A, F) 7,333 fine-mapped for 21 autoimmune diseases from Farh et al.[27], (B, G) 3,768 fine-mapped SNPs for inflammatory bowel disease from Huang et al.[28], (C, H) 1,851 fine-mapped SNPs for 49 traits from UK Biobank[29], (D, I) 1,379 fine-mapped SNPs without functional data for 49 traits from UK Biobank[29], and (E, J) 14,807 GWAS significant SNPs[30,31], from 10 LD-, MAF-, and genomic element-matched control SNPs. We report the average AUPRCs and AUROCs of even/odd-chromosome classifiers. Differences for AUROCs and AUPRCs attained between (1) baseline-LD and baseline-LD+joint model, (2) baseline-LD and baseline-LD+marginal model, and (3) baseline-LD+joint and baseline-LD+marginal were largely significant (p-val < 0.008). Numerical results, including results using the most matched control SNPs (instead of 10), are reported in Supplementary Data 21.

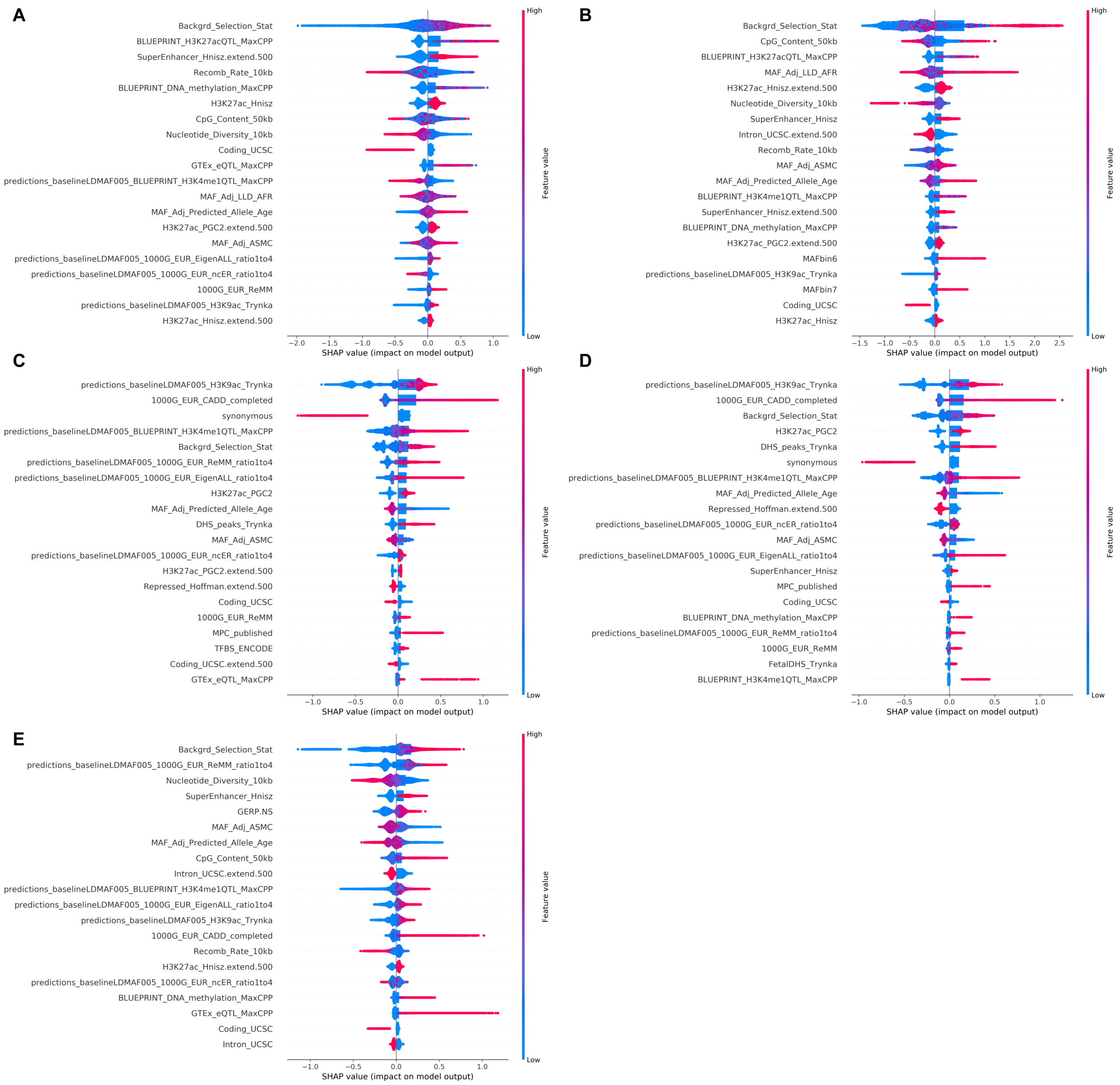

Supplementary Figure 7. Feature importance of baseline-LD+joint model in predicting fine-mapped or GWAS significant SNPs. We applied SHAP[21] to assess which features from the baseline-LD+joint drive the prediction of fine-mapped or GWAS significant SNPs from 10 matched control SNPs for each positive SNP. We report the signed impact of top 20 feature for each of 4 fine-mapped SNPs and GWAS significant SNPs: (A) Farh et al., (B) Huang et al., (C) Weissbrod et al., (D) Weissbrod et al. (fine-mapped without functional data), (E) GWAS significant SNPs. We obtained similar results for even/odd chromosome classifiers; we report odd chromosome results here (see full results online; see URLs).

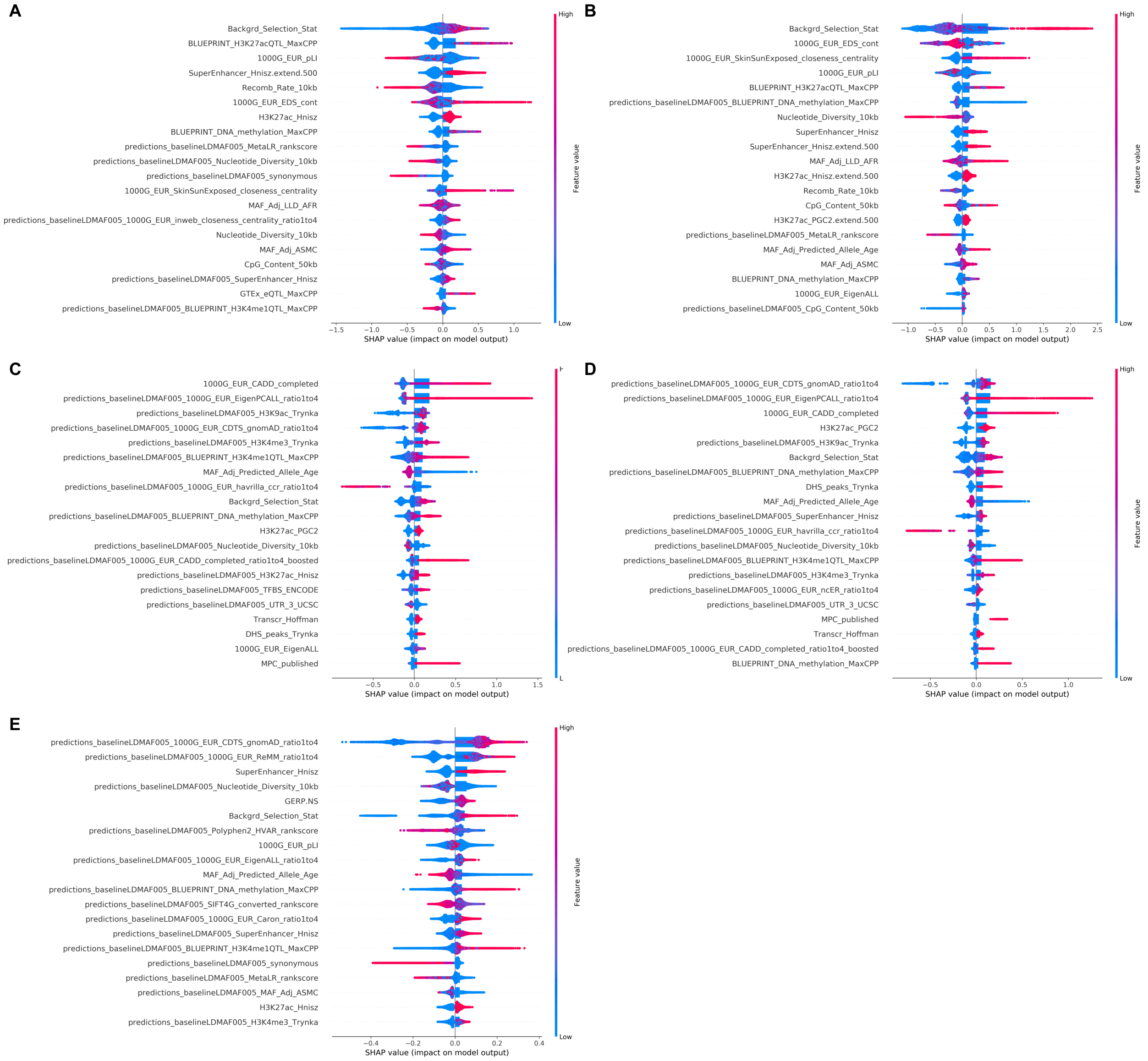

Supplementary Figure 8. Feature importance of baseline-LD+marginal model in predicting fine-mapped or GWAS significant SNPs. We applied SHAP[21] to assess which features from the baseline-LD+marginal drive the prediction of fine-mapped or GWAS significant SNPs from 10 matched control SNPs for each positive SNP. We report the signed impact of top 20 feature for each of 4 fine-mapped SNPs and GWAS significant SNPs: (A) Farh et al., (B) Huang et al., (C) Weissbrod et al., (D) Weissbrod et al. (fine-mapped without functional data), (E) GWAS significant SNPs. We obtained similar results for even/odd chromosome classifiers; we report odd chromosome results here (see full results online; see URLs).

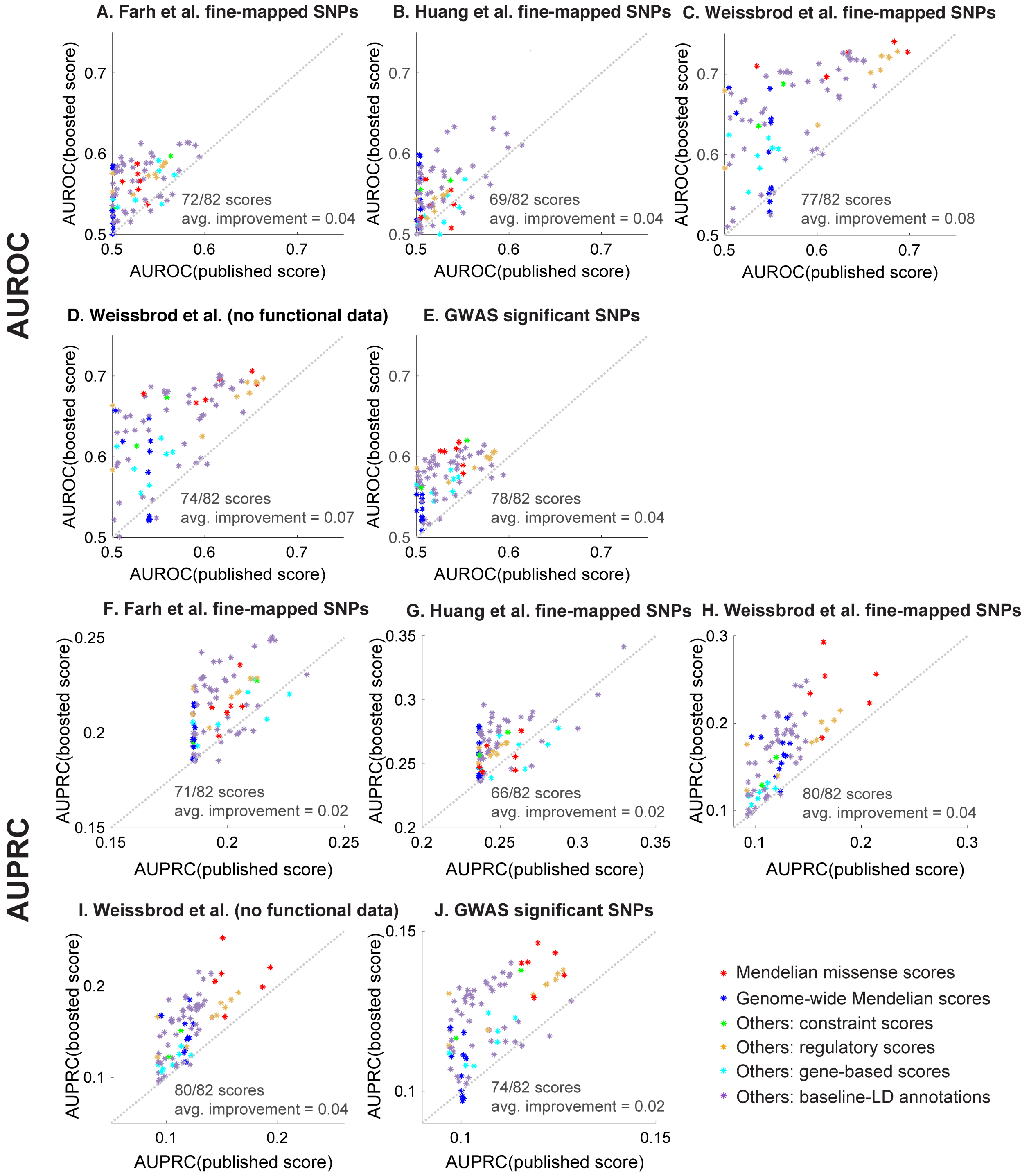

Supplementary Figure 9. Classification of fine-mapped disease SNPs in single-score analysis using published and boosted scores. We report the classification accuracy (AUROC and AUPRC) of each of the 82 boosted scores compared to the corresponding published score. We report AUROC (resp. AUPRC) on (A, F) 7,333 fine-mapped for 21 autoimmune diseases from Farh et al.[27], (B, G) 3,768 fine-mapped SNPs for inflammatory bowel disease from Huang et al.[28], (C, H) 1,851 fine-mapped SNPs for 49 traits from UK Biobank[29], (D, I) 1,379 fine-mapped SNPs without functional data for 49 traits from UK Biobank[29], and (E, J) 14,807 GWAS significant SNPs[30,31] from 10 LD-, MAF-, and genomic element-matched control SNPs. Numerical results, including results using the most matched control SNPs (instead of 10), are reported in Supplementary Data 23.

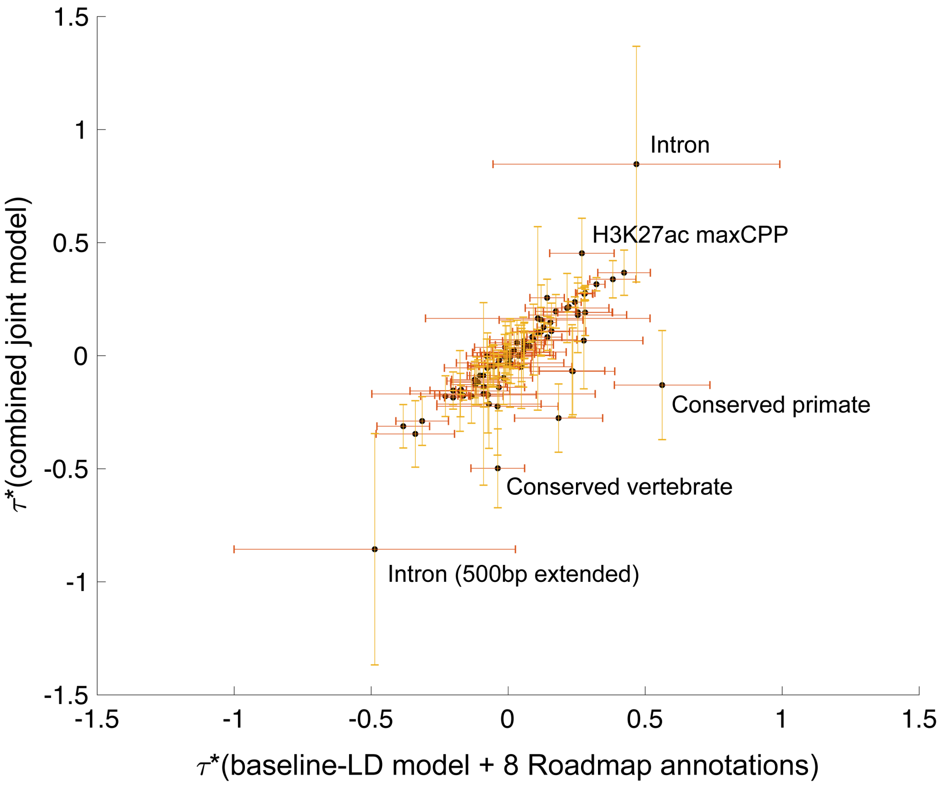

Supplementary Figure 10. Informativeness of the baseline-LD model before and after adding 11 jointly significant binary annotations. We report meta-analyzed τ^* of the baseline-LD model annotations, across 41 independent traits, from two different S-LDSC analyses: (1) the baseline-LD model + 8 Roadmap annotations and (2) the baseline-LD model + 8 Roadmap annotations + 11 jointly significant annotations. Error bars represent 95% confidence intervals. Numerical results are reported in Supplementary Data 25.

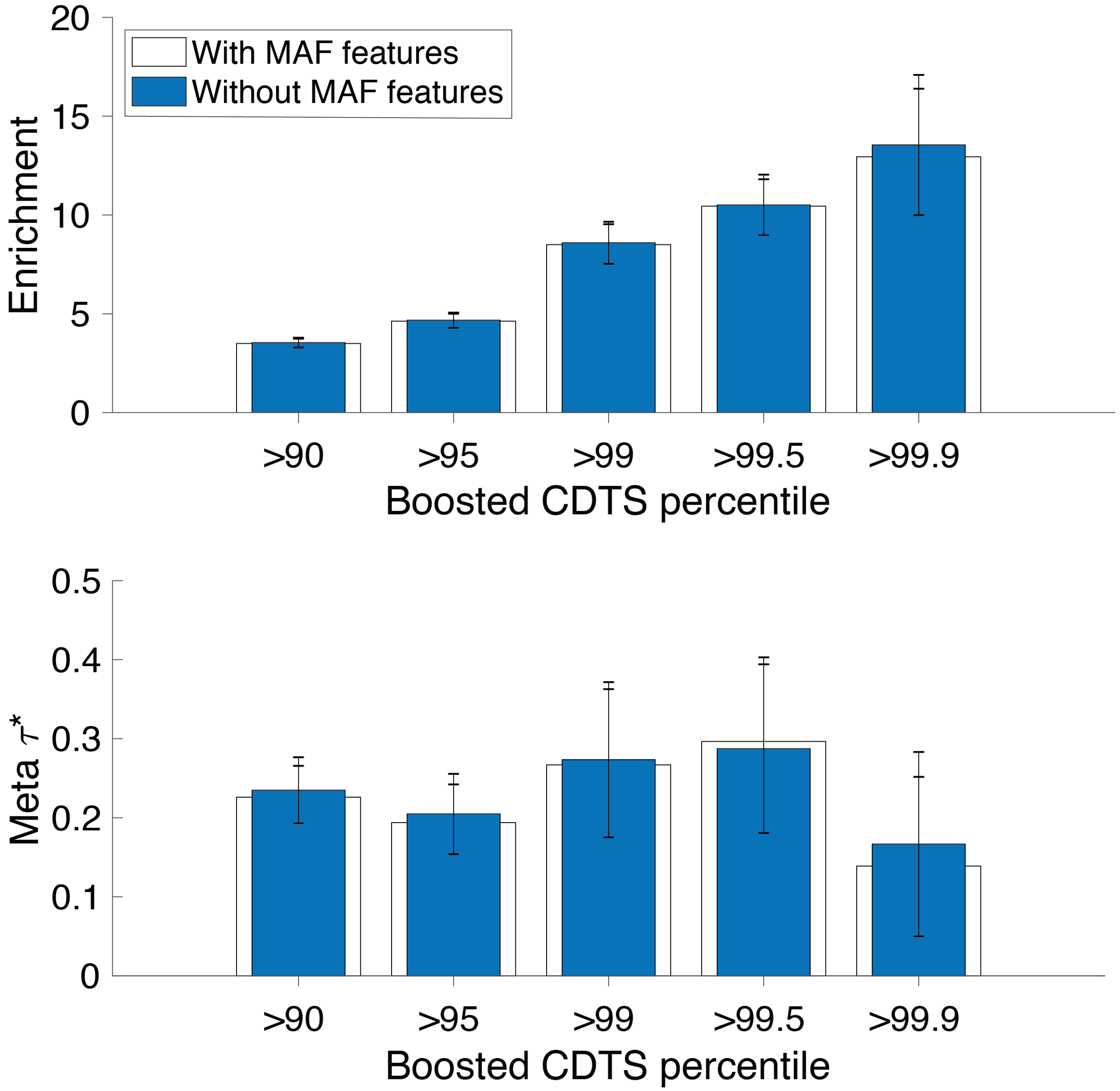

Supplementary Figure 11. Informativeness for common disease of binary annotations derived from boosted CDTS scores with or without MAF features. We applied AnnotBoost to CDTS annotation using all baseline-Ld features and all features excluding MAF bins. Then, we applied S-LDSC, conditioning on published binary CDTS annotations (five thresholds from 90th percentile to 99.9th percentile) and baseline-LD model annotations; and meta-analyzed results across 41 independent traits. We report meta-analyzed enrichments and τ^*. Error bars represent 95% confidence intervals.

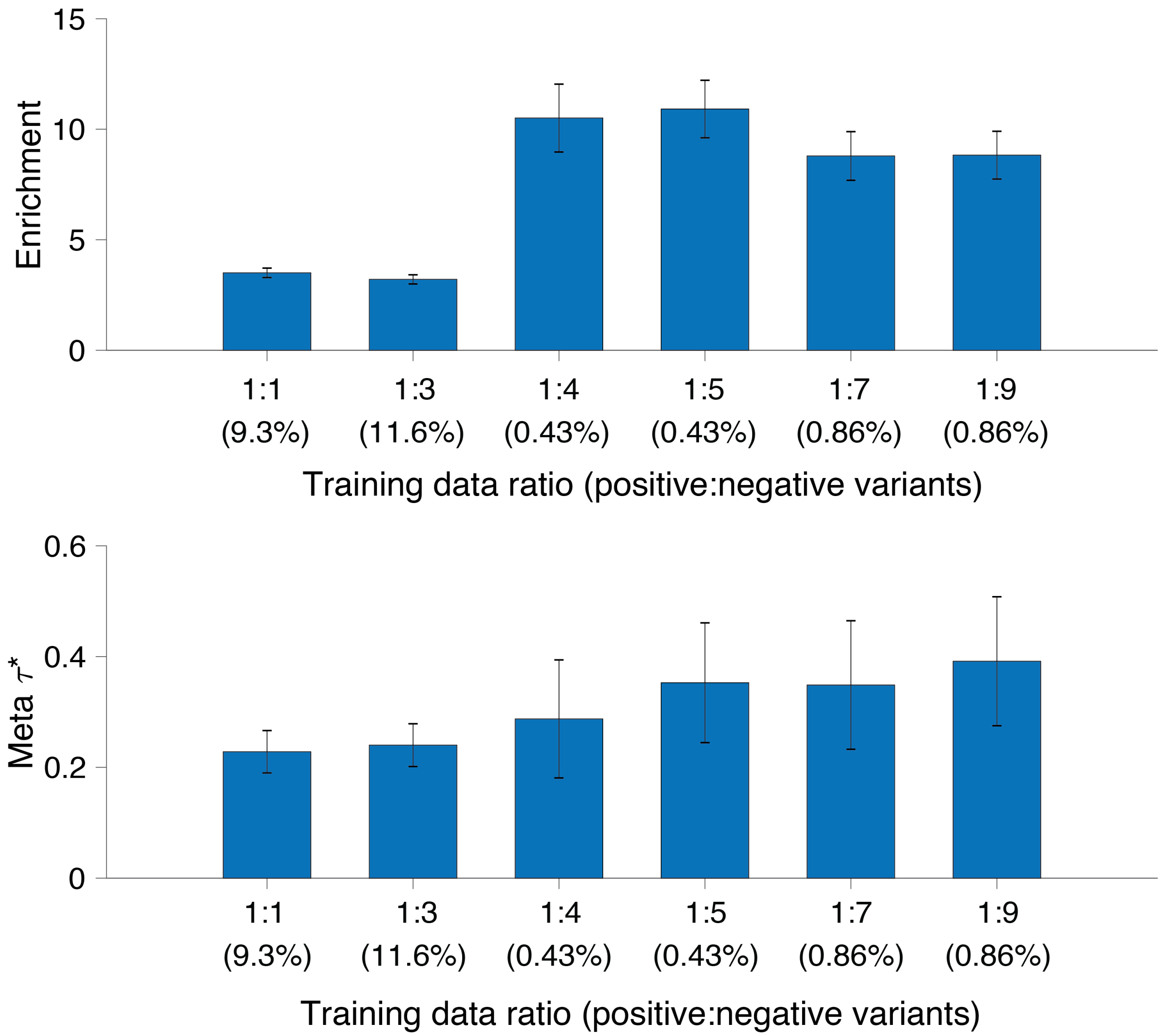

Supplementary Figure 12. Informativeness for common disease of binary annotations derived from boosted CDTS scores using imbalanced data. We applied AnnotBoost to CDTS annotation of varying training data. Then, we applied S-LDSC, conditioning on published binary CDTS annotations (five thresholds from 90th percentile to 99.9th percentile) and baseline-LD model annotations; and meta-analyzed results across 41 independent traits. We report meta-analyzed enrichments and τ^*. Error bars represent 95% confidence intervals.

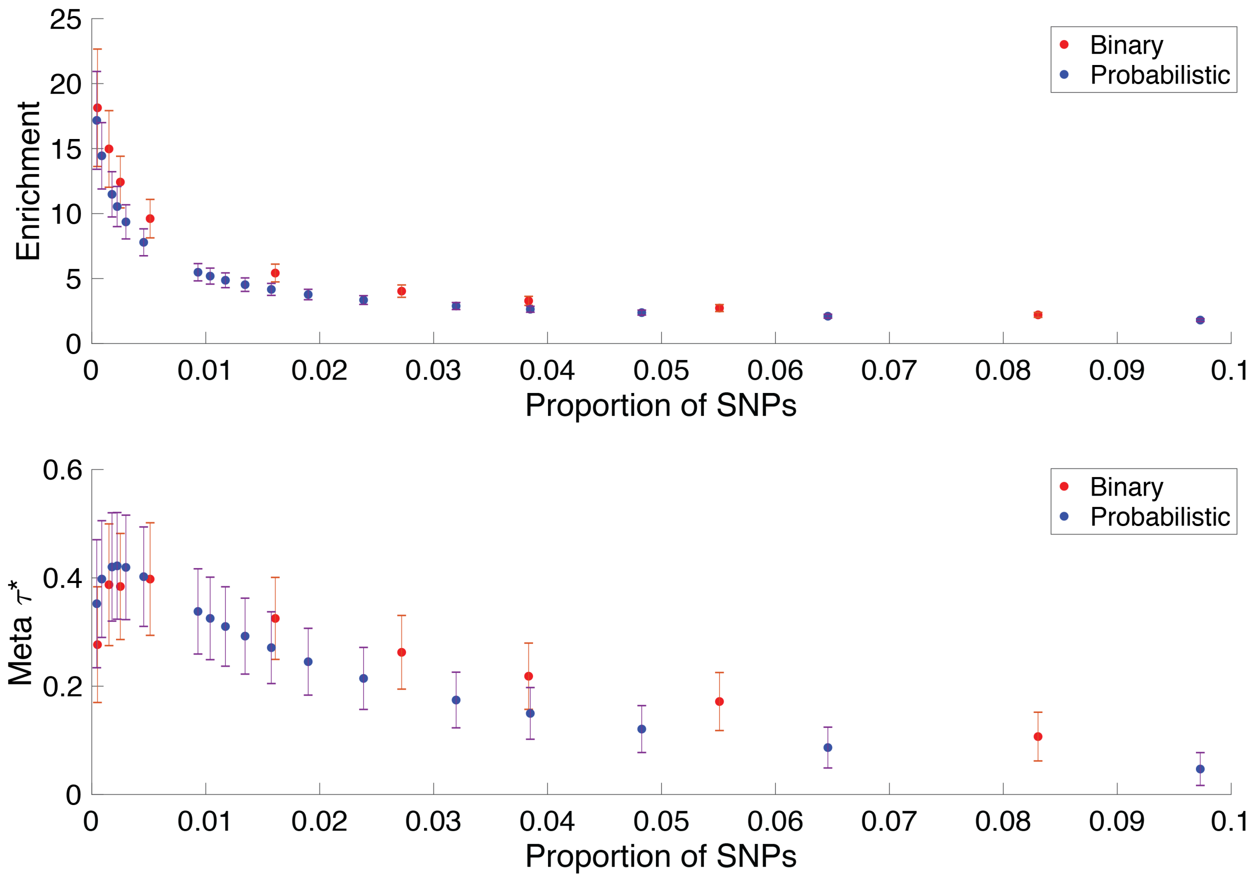

Supplementary Figure 13. Informativeness for common disease of binary and probabilistic annotations derived from published CDTS scores. We constructed binary and probabilistic annotations for published CDTS[32]. We applied S-LDSC, conditional on baseline-LD model annotations and meta-analyzed results across 41 independent traits. We report meta-analyzed enrichments and τ^*. To construct probabilistic annotations of varying proportion of SNPs, we performed the following transformation to upweight the upper percentile and downweight the lower percentile SNPs: $\frac{e^{\alpha * annot}}{e^{\alpha}}$ with α varied from 3 to 2000. Error bars represent 95% confidence intervals.

Supplementary References

1. Hormozdiari, F., Gazal, S., van de Geijn, B., Finucane, H. K., Ju, C. J.-T., Loh, P.-R., Schoech, A., Reshef, Y., Liu, X., O'Connor, L., et al. (2018). Leveraging molecular quantitative trait loci to understand the genetic architecture of diseases and complex traits. Nature Genetics *50*, 1041–1047.

2. Loh, P.-R., Bhatia, G., Gusev, A., Finucane, H. K., Bulik-Sullivan, B. K., Pollack, S. J., de Candia, T. R., Lee, S. H., Wray, N. R., Kendler, K. S., et al. (2015). Contrasting genetic architectures of schizophrenia and other complex diseases using fast variance-components analysis. Nature Genetics *47*, 1385.

3. Loh, P.-R., Kichaev, G., Gazal, S., Schoech, A. P., and Price, A. L. (2018). Mixed-model association for biobank-scale datasets. Nature Genetics *50*, 906–908.

4. Bulik-Sullivan, B., Finucane, H. K., Anttila, V., Gusev, A., Day, F. R., Loh, P.-R., Duncan, L., Perry, J. R., Patterson, N., Robinson, E. B., et al. (2015). An atlas of genetic correlations across human diseases and traits. Nature Genetics *47*, 1236.

5. Barban, N., Jansen, R., De Vlaming, R., Vaez, A., Mandemakers, J. J., Tropf, F. C., Shen, X., Wilson, J. F., Chasman, D. I., Nolte, I. M., et al. (2016). Genome-wide analysis identifies 12 loci influencing human reproductive behavior. Nature Genetics *48*, 1462–1472.

6. Boraska, V., Franklin, C. S., Floyd, J. A., Thornton, L. M., Huckins, L. M., Southam, L., Rayner, N. W., Tachmazidou, I., Klump, K. L., Treasure, J., et al. (2014). A genome-wide association study of anorexia nervosa. Molecular psychiatry *19*, 1085–1094.

7. Jostins, L., Ripke, S., Weersma, R. K., Duerr, R. H., McGovern, D. P., Hui, K. Y., Lee, J. C., Schumm, L. P., Sharma, Y., Anderson, C. A., et al. (2012). Host–microbe interactions have shaped the genetic architecture of inflammatory bowel disease. Nature *491*, 119–124.

8. Allen, H. L., Estrada, K., Lettre, G., Berndt, S. I., Weedon, M. N., Rivadeneira, F., Willer, C. J., Jackson, A. U., Vedantam, S., Raychaudhuri, S., et al. (2010). Hundreds of variants clustered in genomic loci and biological pathways affect human height. Nature *467*, 832–838.

9. Morris, A. P., Voight, B. F., Teslovich, T. M., Ferreira, T., Segre, A. V., Steinthorsdottir, V., Strawbridge, R. J., Khan, H., Grallert, H., Mahajan, A., et al. (2012). Large-scale association analysis provides insights into the genetic architecture and pathophysiology of type 2 diabetes. Nature genetics *44*, 981.

10. Okada, Y., Wu, D., Trynka, G., Raj, T., Terao, C., Ikari, K., Kochi, Y., Ohmura, K., Suzuki, A., Yoshida, S., et al. (2014). Genetics of rheumatoid arthritis contributes to biology and drug discovery. Nature *506*, 376.

11. Okbay, A., Beauchamp, J. P., Fontana, M. A., Lee, J. J., Pers, T. H., Rietveld, C. A., Turley, P., Chen, G.-B., Emilsson, V., Meddens, S. F. W., et al. (2016). Genome-wide association study identifies 74 loci associated with educational attainment. Nature *533*, 539.

12. Cross-Disorder Group of the Psychiatric Genomics Consortium and others. (2013). Identification of risk loci with shared effects on five major psychiatric disorders: a genome-wide analysis. The Lancet *381*, 1371–1379.

13. Schunkert, H., König, I. R., Kathiresan, S., Reilly, M. P., Assimes, T. L., Holm, H., Preuss, M., Stewart, A. F., Barbalic, M., Gieger, C., et al. (2011). Large-scale association analysis identifies 13 new susceptibility loci for coronary artery disease. Nature genetics *43*, 333–338.

14. Schizophrenia Working Group of the Psychiatric Genomics Consortium and others. (2014). Biological insights from 108 schizophrenia-associated genetic loci. Nature *511*, 421–427.

15. Speliotes, E. K., Willer, C. J., Berndt, S. I., Monda, K. L., Thorleifsson, G., Jackson, A. U., Allen, H. L., Lindgren, C. M., Mägi, R., Randall, J. C., et al. (2010). Association analyses of 249,796 individuals reveal 18 new loci associated with body mass index. Nature Genetics *42*, 937–948.

16. Furberg, H., Kim, Y., Dackor, J., Boerwinkle, E., Franceschini, N., Ardissino, D., Bernardinelli, L., Mannucci, P. M., Mauri, F., Merlini, P. A., et al. (2010). Genome-wide meta-analyses identify multiple loci associated with smoking behavior. Nature Genetics *42*, 441.

17. Teslovich, T. M., Musunuru, K., Smith, A. V., Edmondson, A. C., Stylianou, I. M., Koseki,

M., Pirruccello, J. P., Ripatti, S., Chasman, D. I., Willer, C. J., et al. (2010). Biological, clinical and population relevance of 95 loci for blood lipids. Nature *466*, 707.

18. Finucane, H. K., Bulik-Sullivan, B., Gusev, A., Trynka, G., Reshef, Y., Loh, P.-R., Anttila, V., Xu, H., Zang, C., Farh, K., et al. (2015). Partitioning heritability by functional annotation using genome-wide association summary statistics. Nature Genetics *47*, 1228.

19. Gazal, S., Finucane, H. K., Furlotte, N. A., Loh, P.-R., Palamara, P. F., Liu, X., Schoech, A., Bulik-Sullivan, B., Neale, B. M., Gusev, A., et al. (2017). Linkage disequilibrium–dependent architecture of human complex traits shows action of negative selection. Nature Genetics *49*, 1421.

20. Finucane, H. K., Reshef, Y. A., Anttila, V., Slowikowski, K., Gusev, A., Byrnes, A., Gazal, S., Loh, P.-R., Lareau, C., Shoresh, N., et al. (2018). Heritability enrichment of specifically expressed genes identifies disease-relevant tissues and cell types. Nature Genetics *50*, 621.

21. Lundberg, S. M. and Lee, S.-I. (2017). A unified approach to interpreting model predictions. In Advances in Neural Information Processing Systems pp. 4765–4774.

22. Adzhubei, I. A., Schmidt, S., Peshkin, L., Ramensky, V. E., Gerasimova, A., Bork, P., Kondrashov, A. S., and Sunyaev, S. R. (2010). A method and server for predicting damaging missense mutations. Nature Methods *7*, 248.

23. Adzhubei, I., Jordan, D. M., and Sunyaev, S. R. (2013). Predicting functional effect of human missense mutations using polyphen-2. Current Protocols in Human Genetics *76*, 7–20.

24. Kircher, M., Witten, D. M., Jain, P., O'Roak, B. J., Cooper, G. M., and Shendure, J. (2014). A general framework for estimating the relative pathogenicity of human genetic variants. Nature Genetics *46*, 310.

25. Rentzsch, P., Witten, D., Cooper, G. M., Shendure, J., and Kircher, M. (2018). Cadd: predicting the deleteriousness of variants throughout the human genome. Nucleic Acids Research *47*, D886–D894.

26. Havrilla, J. M., Pedersen, B. S., Layer, R. M., and Quinlan, A. R. (2019). A map of constrained coding regions in the human genome. Nature Genetics *51*, 88–95.

27. Farh, K. K.-H., Marson, A., Zhu, J., Kleinewietfeld, M., Housley, W. J., Beik, S., Shoresh, N., Whitton, H., Ryan, R. J., Shishkin, A. A., et al. (2015). Genetic and epigenetic fine mapping of causal autoimmune disease variants. Nature *518*, 337.

28. Huang, H., Fang, M., Jostins, L., Mirkov, M. U., Boucher, G., Anderson, C. A., Andersen, V., Cleynen, I., Cortes, A., Crins, F., et al. (2017). Fine-mapping inflammatory bowel disease loci to single-variant resolution. Nature *547*, 173.

29. Weissbrod, O., Hormozdiari, F., Benner, C., Cui, R., Ulirsch, J., Gazal, S., Schoech, A. P., Van De Geijn, B., Reshef, Y., Marquez-Luna, C., et al. (2020). Functionally-informed fine-mapping and polygenic localization of complex trait heritability. BioRxiv pp. 807792.

30. MacArthur, J., Bowler, E., Cerezo, M., Gil, L., Hall, P., Hastings, E., Junkins, H., McMahon, A., Milano, A., Morales, J., et al. (2016). The new NHGRI-EBI Catalog of published genome-wide association studies (GWAS Catalog). Nucleic Acids Research *45*, D896–D901.

31. Buniello, A., MacArthur, J. A. L., Cerezo, M., Harris, L. W., Hayhurst, J., Malangone, C., McMahon, A., Morales, J., Mountjoy, E., Sollis, E., et al. (2018). The NHGRI-EBI GWAS Catalog of published genome-wide association studies, targeted arrays and summary statistics 2019. Nucleic Acids Research *47*, D1005–D1012.

32. Di Iulio, J., Bartha, I., Wong, E. H., Yu, H.-C., Lavrenko, V., Yang, D., Jung, I., Hicks, M. A., Shah, N., Kirkness, E. F., et al. (2018). The human noncoding genome defined by genetic diversity. Nature Genetics *50*, 333.
