# Supplementary material for: Improving the informativeness of Mendelian disease-derived pathogenicity scores for common disease
Source: Nat Commun. 2020 Dec 7;11:6258. doi: 10.1038/s41467-020-20087-2 (PMC7721881; doi:10.1038/s41467-020-20087-2)
Supplement: Supplementary file 3 — Description of Additional Supplementary Files [file 41467_2020_20087_MOESM3_ESM.docx]

**Description of Supplementary Files**

**File Name: Supplementary Data 1**

**Description:** Informativeness for common disease of binary annotations derived from 11 Mendelian disease-derived missense scores and corresponding boosted scores.

**File Name: Supplementary Data 2**

**Description:** Correlations between published scores and genome-wide boosted scores.

**File Name: Supplementary Data 3**

**Description:** Predictive accuracy of AnnotBoost in out-of-sample predictions of input published scores.

**File Name: Supplementary Data 4**

**Description:** Informativeness for common disease of binary annotations derived from boosted Mendelian disease-derived missense scores, restricted to non-coding regions.

**File Name: Supplementary Data 5**

**Description:** Trait-specific informativeness for common disease of binary annotations.

**File Name: Supplementary Data 6**

**Description:** Heterogeneity of heritability enrichment and τ∗ across traits.

**File Name: Supplementary Data 7**

**Description:** Gene scores derived from published and boosted pathogenicity scores.

**File Name: Supplementary Data 8**

**Description:** 165 reference gene sets of biological importance.

**File Name: Supplementary Data 9**

**Description:** Excess overlap of 165 reference gene sets in each quintile bin of gene scores derived from input pathogenicity scores.

**File Name: Supplementary Data 10**

**Description:** Odds ratios for fold overlaps by gene quintiles from boosted scores and corresponding gene quintiles from published scores in 165 reference gene sets.

**File Name: Supplementary Data 11**

**Description:** Informativeness for common disease of binary annotations derived from 6 genome-wide Mendelian disease-derived scores and corresponding boosted scores.

**File Name: Supplementary Data 12**

**Description:** Informativeness for common disease of binary annotations derived from boosted genome-wide Mendelian pathogenicity scores, restricted to previously unscored variants.

**File Name: Supplementary Data 13**

**Description:** Summary of 18 additional genome-wide scores and 47 baseline-LD model annotations.

**File Name: Supplementary Data 14**

**Description:** Informativeness for common disease of binary annotations derived from 18 additional genome-wide scores + 47 baseline-LD model annotations and corresponding boosted scores.

**File Name: Supplementary Data 15**

**Description:** Informativeness for common disease of binary annotations derived from boosted versions of additional genome-wide scores and baseline-LD model annotations, restricted to previously unscored variants.

**File Name: Supplementary Data 16**

**Description:** Informativeness for common disease of binary annotations derived from 18 additional genome-wide scores + 47 baseline-LD model annotations and corresponding boosted scores without conditioning on 8 Roadmap annotations.

**File Name: Supplementary Data 17**

**Description:** Informativeness for common disease of marginally conditionally significant annotations derived from Mendelian disease-derived missense scores and genome-wide Mendelian disease-derived scores and corresponding boosted scores, when additionally conditioning on 8 Roadmap annotations.

**File Name: Supplementary Data 18**

**Description:** Genome-wide correlations among functional annotations.

**File Name: Supplementary Data 19**

**Description:** Informativeness for common disease of 11 jointly significant binary annotations in a combined joint model.

**File Name: Supplementary Data 20**

**Description:** Evaluation of heritability model fit using loglSS.

**File Name: Supplementary Data 21**

**Description:** Classification of finemapped disease SNPs: multi-score analysis.

**File Name: Supplementary Data 22**

**Description:** Summary of fine-mapped SNPs analyzed and results of single-score classification analysis.

**File Name: Supplementary Data 23**

**Description:** Classification of finemapped disease SNPs: single-score analysis.

**File Name: Supplementary Data 24**

**Description:** Correlation between SLDSC metrics and AUROCs from single-score analysis of classifying fine-mapped SNPs.

**File Name: Supplementary Data 25**

**Description:** Informativeness of the baseline-LD model before and after adding 11 jointly significant binary annotations.
