# Supplementary material for: Improving the informativeness of Mendelian disease-derived pathogenicity scores for common disease
Source: Nat Commun. 2020 Dec 7;11:6258. doi: 10.1038/s41467-020-20087-2 (PMC7721881; doi:10.1038/s41467-020-20087-2)
Supplement: Supplementary file 29 — Reporting Summary [file 41467_2020_20087_MOESM29_ESM.pdf]

nature research

Corresponding author(s): Samuel S. Kim, Alkes L. Price

Last updated by author(s): Oct 1, 2020

Reporting Summary

Nature Research wishes to improve the reproducibility of the work that we publish. This form provides structure for consistency and transparency in reporting. For further information on Nature Research policies, see our Editorial Policies and the Editorial Policy Checklist.

Statistics

For all statistical analyses, confirm that the following items are present in the figure legend, table legend, main text, or Methods section.

n/a	Confirmed	
☐	☒	The exact sample size (n) for each experimental group/condition, given as a discrete number and unit of measurement
☐	☒	A statement on whether measurements were taken from distinct samples or whether the same sample was measured repeatedly
☐	☒	The statistical test(s) used AND whether they are one- or two-sided<br>*Only common tests should be described solely by name; describe more complex techniques in the Methods section.*
☒	☐	A description of all covariates tested
☐	☒	A description of any assumptions or corrections, such as tests of normality and adjustment for multiple comparisons
☐	☒	A full description of the statistical parameters including central tendency (e.g. means) or other basic estimates (e.g. regression coefficient) AND variation (e.g. standard deviation) or associated estimates of uncertainty (e.g. confidence intervals)
☐	☒	For null hypothesis testing, the test statistic (e.g. F, t, r) with confidence intervals, effect sizes, degrees of freedom and P value noted<br>*Give P values as exact values whenever suitable.*
☒	☐	For Bayesian analysis, information on the choice of priors and Markov chain Monte Carlo settings
☒	☐	For hierarchical and complex designs, identification of the appropriate level for tests and full reporting of outcomes
☐	☒	Estimates of effect sizes (e.g. Cohen's d, Pearson's r), indicating how they were calculated

Our web collection on statistics for biologists contains articles on many of the points above.

Software and code

Policy information about availability of computer code

Data collection	n/a; we did not collect data; we analyzed raw genotype-phenotype data from UK Biobank (application 16549). Summary statistics are available at https://alkesgroup.broadinstitute.org/LDSCORE/independent_sumstats/
Data analysis	Our ldsc software (v.1.0.1) is available at http://www.github.com/bulik/ldsc.<br>Our baseline-LD (v.2.1) model annotations are available at https://alkesgroup.broadinstitute.org/LDSCORE/<br>For existing variant-level pathogenicity scores, we used dbNSFP software (v.4.0; https://sites.google.com/site/jpopgen/dbNSFP) to query missense 11 Mendelian missense scores, CADD (v.1.5; https://cadd.gs.washington.edu/download), Eigen/Eigen-PC (v.1.1; http://www.columbia.edu/~ii2135/download.html), ReMM (v.0.3.1; https://charite.github.io/software-remm-score.html), NCBoost (v.1.0.0; https://github.com/RausellLab/NCBoost), ncER (v.1.0; https://github.com/TelentiLab/ncER_datasets), CDTS (no separate version information; http://www.hli-opendata.com/noncoding/), CCR (no separate version information; https://github.com/quinlan-lab/ccr), DeepSEA (v.2018; https://hb.flatironinstitute.org/deepsea/). Analyzed gene-level scores come from the following sources: DIS (Table S1 in PMC6758908), pLI (https://gnomad.broadinstitute.org/downloads), LIMBR (Table S1 in PMC6369453), network connectivity scores (https://alkesgroup.broadinstitute.org/LDSCORE/Kim_pathwaynetwork/), EDS (Table S1 in PMC7010980).<br><br>All new annotations analyzed in this work with a concise tutorial is available at https://alkesgroup.broadinstitute.org/LDSCORE/Kim_annotboost/

For manuscripts utilizing custom algorithms or software that are central to the research but not yet described in published literature, software must be made available to editors and reviewers. We strongly encourage code deposition in a community repository (e.g. GitHub). See the Nature Research guidelines for submitting code & software for further information.

Data

Policy information about availability of data

All manuscripts must include a data availability statement. This statement should provide the following information, where applicable:

- Accession codes, unique identifiers, or web links for publicly available datasets
- A list of figures that have associated raw data
- A description of any restrictions on data availability

The Data availability section includes URL links for all publicly available datasets analyzed throughout the paper, as well as new annotations and method generated.

Field-specific reporting

Please select the one below that is the best fit for your research. If you are not sure, read the appropriate sections before making your selection.

[x] Life sciences [ ] Behavioural & social sciences [ ] Ecological, evolutionary & environmental sciences

For a reference copy of the document with all sections, see nature.com/documents/nr-reporting-summary-flat.pdf

Life sciences study design

All studies must disclose on these points even when the disclosure is negative.

Sample size	We used the larger sample size that was available (the same set of traits analyzed in Hormozdiari et al. Nature Genetics 2018). These set of traits are based on datasets with sufficient sample size, SNP heritability and polygenicity (z score of total SNP heritability estimated by stratified LD score regression).
Data exclusions	Our study was restricted to data sets of European ancestry. We excluded the HLA regions and only analyzed autosomes.
Replication	No replication dataset was analyzed as our analyses are already based on a meta-analyses of 41 independent traits.
Randomization	We performed no randomization and analyzed all individuals for the GWAS studies analyzed.
Blinding	We did not collect data for the study and used publicly available GWAS data and the UK Biobank data.

Reporting for specific materials, systems and methods

We require information from authors about some types of materials, experimental systems and methods used in many studies. Here, indicate whether each material, system or method listed is relevant to your study. If you are not sure if a list item applies to your research, read the appropriate section before selecting a response.

Materials & experimental systems

n/a	Involved in the study	
[x]	[ ]	Antibodies
[x]	[ ]	Eukaryotic cell lines
[x]	[ ]	Palaeontology and archaeology
[x]	[ ]	Animals and other organisms
[x]	[ ]	Human research participants
[x]	[ ]	Clinical data
[x]	[ ]	Dual use research of concern

Methods

n/a	Involved in the study	
[x]	[ ]	ChIP-seq
[x]	[ ]	Flow cytometry
[x]	[ ]	MRI-based neuroimaging
